# Comparing the First-Pass Success Rate of the King LTS-D and the i-gel Airway Devices in Out-of-Hospital Cardiac Arrest

**DOI:** 10.7759/cureus.30987

**Published:** 2022-11-01

**Authors:** Patrick Price, Anne Laurie, Eric Plant, Kavish Chandra, Tushar Pishe, Keith Brunt

**Affiliations:** 1 Medicine, Dalhousie Medicine New Brunswick, Saint John, CAN; 2 Education, Concordia University, Montreal, CAN; 3 Emergency Medicine, Dalhousie University, Halifax, CAN; 4 Emergency Medicine, Saint John Regional Hospital/Horizon Health Network, Saint John, CAN

**Keywords:** emergency medical services, pre-hospital, emergency medicine, paramedic, supraglottic airway

## Abstract

Objectives: Significant heterogeneity exists internationally in the airway devices used in the pre-hospital setting during cardiac arrest. This study evaluated the first-pass success (FPS) rate of two supraglottic airways (SGAs) used by paramedics during out-of-hospital cardiac arrest: the King LTS-D and the i-gel.

Methods: By examining 2,680 patient care records compiled by Ambulance New Brunswick between 2015 and 2020, we evaluated the FPS rate of the two SGAs using a 2x2 Pearson chi-square test for association, and a Mann-Whitney U test, to determine whether there were significant differences in FPS rates.

Results: Our study demonstrated a statistically significant association between airway devices and FPS favoring the i-gel with an FPS of 90.6% compared to a 76.6% FPS with the King LTS-D; *X*^2^(1) = 96.01, p < 0.001. The odds of successfully inserting the airway on the first attempt were 2.94 times higher if paramedics used the i-gel than if they used the King LTS-D with a 95% CI [2.32, 3.60]. Mann-Whitney’s U test for SGA differences favored the i-gel in fewer attempts for successful insertion (z = -4.357, p < 0.001, d = 0.15).

Conclusions: Among patients in New Brunswick with out-of-hospital cardiac arrest, paramedics had a higher FPS rate with the i-gel compared to the King LTS-D. Our study also found a statistically significant difference between the King LTS-D and i-gel, favoring the i-gel in fewer attempts. Our findings suggest that the i-gel provides a more consistent FPS rate compared to the King LTS-D within our study populations; however, further research is necessary to determine the clinical implications of this. While multiple attempts at tracheal intubation are associated with negative clinical outcomes, no such evidence exists for SGAs.

## Introduction

Optimal airway management is an important topic in medical research. Despite the emphasis, it is difficult to evaluate the effectiveness of airway devices, particularly those used outside a controlled hospital environment [1-3]. Factors such as provider experience and training, etiology of respiratory distress, environmental factors, confounding variables, and a lack of consensus on which outcomes to measure have led to a noticeable diversity in airway policy and procedure globally [3-5]. Additional research is required to determine optimal airway interventions. As such, we conducted this study to evaluate the first-pass success (FPS) rate of two supraglottic airway (SGA) devices during out-of-hospital cardiac arrest (OHCA) used by paramedics in New Brunswick, Canada. This research has been previously presented as a poster presentation at the Canadian Association of Emergency Physicians' Annual Conference on May 30, 2022.

Background

Significant heterogeneity exists internationally in the airway devices used in the pre-hospital setting during cardiac arrest [3-6]. Multiple factors influence the choice and provision of airway devices, including ease of placement, speed of placement, skill required for placement, complication rates, and cost and supply chain management [7]. Quality care improvement requires assessment of both procedure and equipment selection to impact the standard of care for patients, as the successful use and application of airway devices increase patient survivability [4-9].

Historically, an endotracheal tube was considered the gold standard for airway management during resuscitation [10,11]. More recent research points to the important role supraglottic airway devices can play in advanced airway management [2-4,12]

Ideally, institutional policy surrounding the choice and availability of airway devices in the pre-hospital setting is guided by evidence obtained through large and randomized controlled trials [8]. This type of evidence is currently limited, and as such, there is no consensus on the optimal airway device for pre-hospital clinicians during resuscitation attempts [2-4,12]. There is, however, a consensus that during cardiopulmonary resuscitation (CPR), interruptions to compressions for airway insertion should be no greater than 10 to 15 seconds [2,12].

Some literature suggests that some historical data regarding endotracheal tube effectiveness in out-of-hospital cardiac arrest may be biased [10,13]. One potential problem is that, in the context where providers started with endotracheal intubation, are unsuccessful, and place a supraglottic airway device as a “rescue device” [3,10,11]. This delay in establishing an advanced airway may contribute to deleterious patient outcomes and influence data [3,10,11]. Endotracheal intubation (ETI), in general, also typically takes longer than the placement of an SGA [6,13,14]. This delay in ventilation and disruption of chest compressions may cause hypoxia, resulting in decreased cerebral and myocardial oxygenation, and may lead to negative clinical outcomes [2,10].

The previously held notion that “endotracheal intubation is always best” is an oversimplification [4,10]. For example, one 2001 study showed that endotracheal tubes may be improperly positioned more often than is reflected in the literature due to inadequate verification methods for out-of-hospital intubation [5]. Multiple studies indicate that even with skilled providers, tracheal intubation during out-of-hospital cardiac arrest may take longer, be more error-prone, and result in negligible clinical improvements for patients who survive hospital discharge [10,11]. The AIRWAYS-2 trial, a large randomized controlled trial, showed initial ventilation success with endotracheal tubes at 79%, whereas the supraglottic airway device group had 87.4% [4]. This study also showed no statistically significant difference between tracheal intubation and supraglottic airway device regarding modified Rankin score in patients at the time of discharge [4].

Insertion of a supraglottic airway device is more straightforward and consistently requires less time than an endotracheal tube [3,4,14]. The use of SGAs requires less training and is an easier skill to maintain [3,4]. A randomized clinical trial by Wang and his colleagues (2018) showed significantly greater 72-hour survival in patients who experienced out-of-hospital cardiac arrest and received an SGA instead of an endotracheal tube for airway management [3]. The latest Advanced Cardiovascular Life Support (ACLS) guidelines often encourage initial airway management in cardiac arrest with SGAs [2,12]. With the role of SGAs in an out-of-hospital cardiac arrest being clearly identified in the literature; it is imperative to establish which of the large variety of SGAs is most effective [6].

The King LTS-D is a laryngeal tube airway that is made up of a plastic lumen with two balloon cuffs. The oropharyngeal cuff is positioned near the middle of the tube. It is larger and is designed to prevent retrograde air leaks during ventilation. The smaller cuff is the esophageal cuff, which is intended to prevent air from being diverted into the esophagus and stomach. The tube has fenestrations between the two balloon cuffs that allow for the free passage of air towards the trachea. There is a gastric access lumen that allows for passage of an orogastric tube.

The i-gel airway is a second-generation supraglottic airway. It consists of a hardened plastic lumen that is ovoid in shape. It has a gel-like cuff at its distal end that blocks the esophagus and directs air towards the trachea. This cuff does not require inflation. The i-gel also has a port designed for orogastric tube insertion.

While there is some limited data available comparing the two devices, there is minimal research about ease of use and FPS rate [15]. With current resuscitation guidelines emphasizing high-quality CPR with limited interruptions, it is important to identify the device that offers the most straightforward and reliable application.

## Materials and methods

This study was based in New Brunswick, a 72,908 km² province with a population of 775,610 inhabitants on Canada’s eastern coast. This research was reviewed and approved by the research ethics board at Horizon Health Network in Saint John, New Brunswick on October 14th, 2020, file number 100995. This approval was granted with a waiver of informed consent for the secondary use of de-identified data. Further approval to use their extant data was obtained from the operators of Ambulance New Brunswick (ANB; Medavie Health Services Inc., NB). This study complies with the STROBE (Strengthening the Reporting of Observational Studies in Epidemiology) recommendations for reporting observational cohort studies.

Current policy dictates that paramedics at Ambulance New Brunswick (ANB) have several options for initiating and maintaining an airway, the most invasive of which is the SGA. There have been two SGAs available at different times at ANB. The King LTS-D supraglottic airway was introduced in December 2008 and it was replaced by the i-gel supraglottic airway device in December 2017. 

This study has a retrospective comparative cross-sectional design and was conducted using patient care records compiled by ANB between February 1, 2015 and September 30, 2020. This selection of patient care records contains exclusively patients that experienced out-of-hospital cardiac arrest across New Brunswick during the identified timespan. The database is compiled by ANB staff as part of their policy to audit every cardiac arrest call. Although paramedics may use advanced airways in settings outside of cardiac arrest, this data set deals exclusively with out-of-hospital cardiac arrest and subsequent airway management. Cardiac arrest was defined as [pulseless on the scene or en route to the hospital, and resuscitative efforts were started/attempted] via ANB policy. Cardiac arrest could have been of any etiology, including those induced by trauma. 

 The primary outcome was to identify the FPS rate of airway insertion between two SGAs: the King LTS-D and the i-gel. This will determine whether one SGA has an FPS advantage over the other SGA. Successful airway placement was determined clinically by paramedics as evidenced by end-tidal capnography, auscultation and visual confirmation of chest movements with ventilation. The secondary outcome was to determine whether there were differences between the number of insertion attempts to successful insertion between the King LTS-D and the i-gel and whether any differences were statistically significant. This will determine whether one SGA had a lower number of insertion attempts within our study populations. 

**Figure 1:**
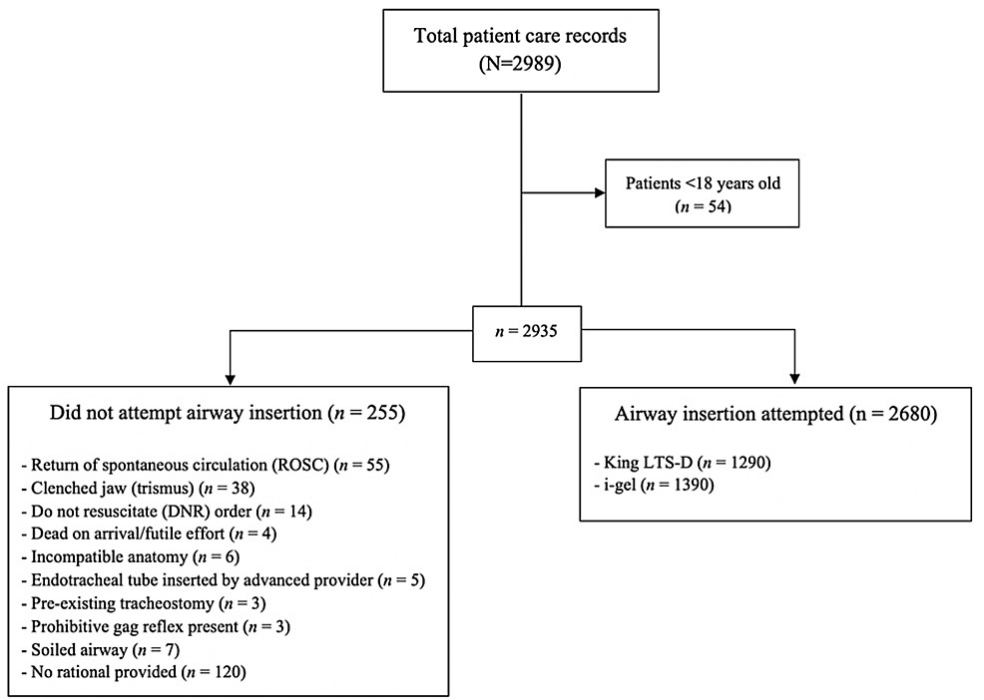
Flow of study patients

The original data set includes both patient and non-patient factors. Patient factors include heart rate, oxygen saturation, sex, age and weight. Non-patient factors were: if an advanced airway was attempted, the size of the airway used, if the placement of the airway was successful, a number of attempts at airway insertion, if an orogastric tube was inserted, if the airway was in place at the end of the call, and a section for comments. The data set contained 1,472 entries between February 1, 2015 and November 30, 2017 during which time the King LTS-D was being used, and 1517 entries between December 1, 2017 and September 30, 2020 while the i-gel was being used. Data were refined further to remove any entries that: involved patients under the age of 18 (n = 54) or that records indicated "no attempt was made" at airway insertion (n = 255). The rational provided for not attempting airway insertion in the 255 cases can be summarized within the following categories: return of spontaneous circulation (ROSC) (n = 55); clenched jaw (trismus) (n = 38); do not resuscitate (DNR) order (n = 14); dead on arrival/futile effort (n = 4); incompatible anatomy (n = 6); endotracheal tube inserted by the advanced provider (n = 5); pre-existing tracheostomy (n = 3); prohibitive gag reflex present (n = 3); soiled airway (n = 7) and no rational provided (n = 120). The final sample for analysis was N = 2,680 (King LTS-D, n = 1,290; i-gel, n = 1,390). The selection and exclusion criteria are presented as a consort diagram in Figure 1.

The age and sex characteristics of study participants can be found in Table 1, which demonstrates that the mean ages and gender across both SGAs are very similar to one another. In the software Statistically Package for Social Sciences (SPSS) (version 28) a variable for FPS was created by coding cases as 1 that were 1) successful and 2) successful on the first attempt. Thus, all other cases indicated as either unsuccessful or successful after more than one attempt were coded as 0.

**Table 1:** Patient demographics by supraglottic airway

Airway Device	Age in Years	Sex		Total (%)
	Mean (SD)	Male (%)	Female (%)	
King LTS-D	64.75 (15.73)	844 (65.4%)	446 (34.6%)	1,290 (48.1%)
i-gel	64.96 (15.12)	968 (69.6%)	422 (40.4%)	1,390 (51.9%)
Total	64.86 (15.41)	1,812 (67.6%)	868 (32.4%)	2,680 (100%)

Subsequently, a 2x2 Pearson chi-square test for association was conducted to identify an association between the FPS (yes or no) and the type of SGAs (King LTS-D and i-gel), with an alpha level set to 0.05. The effect size, in the form of an odds ratio (with 95% CIs), will be reported. As both independent variables were categorical and the expected cell frequencies were greater than five, the chi-square assumptions were met.

For the second research question, we explored whether there were differences between the number of attempts (dependent variable) for both SGAs (independent variable), with an alpha level set to 0.05, and the effect size in the form of Cohen’s D (with 95% CIs) performed through G*Power [16]. We removed all cases where paramedics reported they were not successful in inserting the SGAs regardless of the number of attempts. There were 177 cases of the King LTS-D and 46 cases of the i-gel that were unsuccessful (n = 216) leaving a total of 1,117 King LTS-D instances and 1,347 i-gel instances (N = 2,464). We examined the raw frequencies of attempts to successful insertions for both SGAs, then compared the groups for any statistically significant group differences. 

## Results

Research question 1 

What is the FPS rate of the King LTS-D and i-gel?

**Figure 2:**
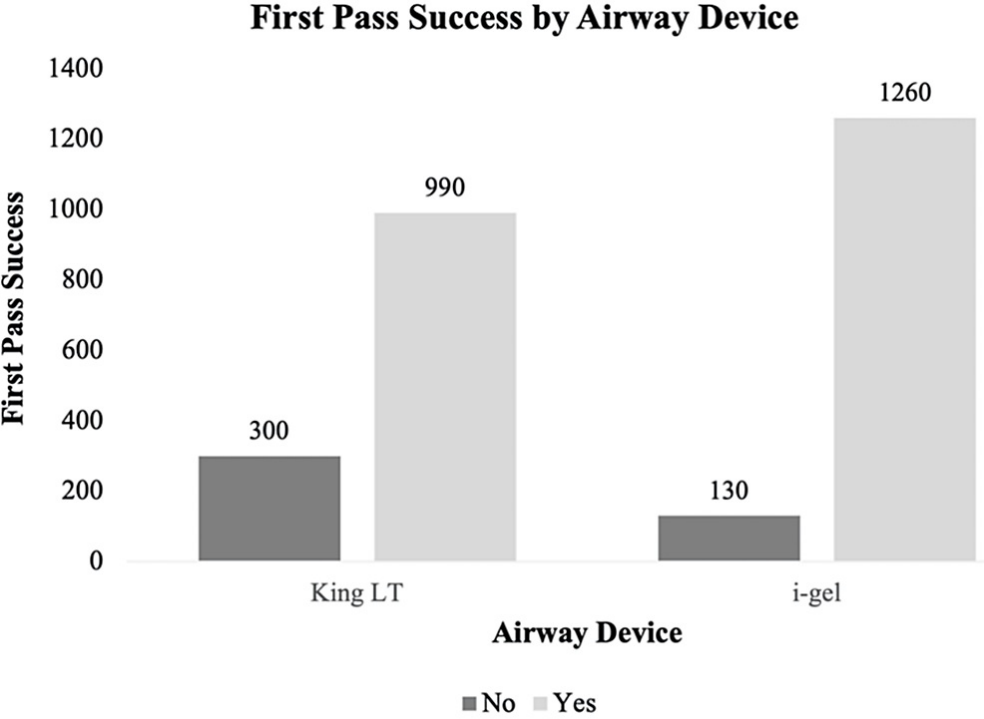
Supraglottic airway comparison

The FPS rate was 76.7% for the King LTS-D and 90.6% for the i-gel, therefore, the i-gel resulted in an overall higher percentage of FPS compared with the King LTS-D. Figure 2 highlights the differences between the two SGAs by looking at the raw frequencies for the first pass to successful insertion. There was a statistically significant association between airway device and FPS, X^2^(1) = 96.01, p < 0.001. The odds of successfully inserting the airway on the first attempt were 2.94 times higher if paramedics used the i-gel than if they used the King LTS-D, OR = 2.94, p < 0.001 (95% CI: 2.35, 3.67).

Research question 2 

Are there differences in the number of attempts at successful airway placement by airway device?

We ran a partial correlation controlling for both age and sex to determine the association between the number of airways attempts to successful insertion and airway type. There was a small but significant correlation (r = -0.093, p < 0.001) possibly suggesting that the number of attempts to successful insertion is lower in the i-gel than that of the King LTS-D. The raw frequencies in Table 2 indicate that the i-gel had fewer number of attempts for successful insertion than the King LTS-D, despite having a larger sample size. This was confirmed when exploring the mean number of attempts for both SGAs. The i-gel had a lower number of insertion attempts (Mean = 1.07, SD = 0.29) than the King LTS-D (Mean= 1.14, SD = 0.44). To test whether these results were statistically significant, we first tested our data to see if they respected the necessary conditions to perform parametric tests. The assumption of normality was violated as the data were rightly skewed due to a large number of FPSs. Therefore, we continued with the non-parametric Mann-Whitney U test. The Mann-Whitney U test indicated a statistically significant difference in the mean rank number of attempts for successful insertion favouring a lower mean rank for the i-gel (Median = 1, Mean Rank = 1,204.75) than for the King LTS-D (Median = 1; Mean Rank = 1,265.97), U = 714,918.00, z = -4.357, p < 0.001, d = 0.15.

**Table 2:** Number of attempts to successful insertion per supraglottic airway ^a^Number of attempts leading to successful insertion of SGA.

Number of Attempts^a^	King LTS-D (Total %)		i-gel (Total %)
1	990 (88.6%)		1,260 (93.5%)
2	100 (9.0%)		75 (5.6%)
3	23 (2.1%)		12 (0.9%)
4	3 (0.3%)		0 (0%)
5	1 (0.1%)		0 (0%)
Total N	1117		1,347

## Discussion

Our study shows that the i-gel device has a 2.94 times higher FPS rate when compared to the King LTS-D when used by New Brunswick paramedics during out-of-hospital cardiac arrest. There was also a statistically significant difference between the two SGAs regarding their mean rank of attempts for successful placement, favoring the i-gel with a lower mean rank number of attempts. Regarding the differences in the number of attempts between the two SGAs, a small effect size (d = 0.15) was expected with our large sample size as the variability of effect sizes decreases with increasing sample size [17]. Such a large sample size provides a better indication of real-world effects, and as such we suggest that the effect size is due to differences in SGAs and not by random effects [17]. Therefore, the results of our research questions lead us to argue for the continuation of the i-gel by paramedics when compared with the King LTS-D. 

The environment in which our study participants inserted the SGAs is varied. Patients are not encountered in standardized settings in the pre-hospital environment [8,9]. This pre-hospital setting does not allow for the same pre-procedure preparation and control as the operating room or even the emergency department [9]. Providers in the pre-hospital environment often do not have the benefit of optimal positioning, lighting or support personnel [8,9]. SGAs have been shown to have a higher FPS rate in OHCA compared to ETI [4]. A growing body of literature suggests that in the unpredictable conditions in which pre-hospital clinicians initiate their airway insertions, easy-to-use airway management tools like SGAs are considered to have an advantage over ETI [2,12,18-20]. Therefore, there is a clinical imperative to determine which of the wide variety of available SGAs provides the best usage characteristics.

Research published in 2018 showed that the average time for placement by paramedic students in a “simulated tactical environment” on high-fidelity manikins was 39.7 seconds with the King LTS-D versus 14.4 seconds with the i-gel [15]. The most recent ACLS guidelines emphasize the importance of high-quality chest compressions and limited interruptions [2,12]. Our study, as well as the corresponding literature review, suggest that using an SGA with a higher FPS rate and a lower time to successful insertion is compatible with the current ACLS guidelines.

A high FPS is desirable in the setting of advanced airway management [18-20]. Multiple attempts at tracheal intubation have been associated with negative patient outcomes, including aspiration, hypoxemia, and cardiac arrest [18-20]. A study of out-of-hospital cardiac arrests in the emergency department from 2014 suggests that FPS failure during endotracheal intubation was associated with lower frequencies of ROSC [20]. To our knowledge, there is no research on the clinical implications of FPS with SGAs. Our data set did not contain any information on patient outcomes and as such, we were unable to investigate that relationship. Randomized controlled trials comparing airway devices are rare [2,3,12]. There is a lack of consensus on what to measure, as well as limited ability to conduct these studies safely [2,12]. Most of the comparisons of airway devices use manikins, occur in the setting of in-hospital general anesthesia, or are observational studies with acknowledged limitations [8,21].

While certain inferences can be made from our results, they must be interpreted with caution. Ultimately the best airway device is not one that has low training requirements or is easy to insert. The best airway device is one that reliably provides ventilation and oxygenation for patients, protects the airway from aspiration, and facilitates better outcomes such as the return of spontaneous circulation, survival to hospital admission and discharge, and improved neurological outcomes [4,10,12]. Different providers have different performance characteristics and describing one airway as superior to another based solely on FPS rate is an oversimplification [21,22].

This study used exclusively extant data provided by ANB. As such, the data were not compiled with our research question in mind. Some exclusions may have been avoided with more standardized or consistent documentation. Our data set was reliant on self-report by paramedics, and this may impact inter-rater reliability. Because of the way the data were collected and compiled, it made a reliability check impossible.

Another limitation was the period for which we evaluated these devices. The King LTS-D had been used for 10 years prior to the sample period. This would allow for increased practice with the device. Effectively, we are comparing the period for which paramedics have the most practice (with the King LTS-D) with the period in which paramedics have the least practice (with the i-gel). Studies suggest that competency with SGAs is improved with practice [8,14,23].

Paramedics in New Brunswick have a heterogeneous frequency with which they encounter out-of-hospital cardiac arrest. Rural paramedics in sparsely populated areas may go over a year without the need to use a supraglottic airway. We were unable to control for the geographic location of cardiac arrest or the frequency with which individual paramedics used the SGAs. Had we been able to control for this-we anticipate we would have seen geographical variability.

## Conclusions

Among patients in New Brunswick with out-of-hospital cardiac arrest, paramedics had a higher FPS rate with the i-gel compared to the King LTS-D. Our study also found that there was a statistically significant difference between the number of attempts to successfully insert the two SGAs, favoring the i-gel. Our findings suggest that the i-gel provides a more reliable first-pass insertion compared to the King LTS-D within our study populations. With an increasing body of evidence to suggest that SGAs should be used as the initial airway of choice for OHCA, a clear clinical imperative to determine which of the many devices currently available provides clinicians and patients with the most favorable usage characteristics is evident. Further research comparing SGAs is warranted to evaluate these nuances.
